# Psychedelics Reverse the Polarity of Long-Term Synaptic Plasticity in Cortical-Projecting Claustrum Neurons

**DOI:** 10.1523/ENEURO.0047-25.2025

**Published:** 2025-10-23

**Authors:** Tanner L. Anderson, Artin Asadipooya, Pavel I. Ortinski

**Affiliations:** Department of Neuroscience, College of Medicine, University of Kentucky, Lexington, Kentucky 40536

**Keywords:** 5-HT2A, claustrum, long-term potentiation, psychedelics, spike-timing–dependent plasticity, synaptic plasticity

## Abstract

Psychedelic drugs have garnered increasing attention for their therapeutic potential in treating a variety of psychiatric diseases, such as depression, anxiety, and substance use disorder. The claustrum (CLA), a brain area with remarkable interconnectivity to frontal cortices, has recently been shown to have a dense population of serotonin 2 receptors (5-HT2Rs) that are activated by psychedelics. Because psychedelic therapy can require as little as one treatment session, it has been speculated that psychedelics achieve their long-term remedial effects by inducing neuroplasticity in brain areas responsible for psychiatric disease states, such as the anterior cingulate cortex (ACC). However, the effects of psychedelics on synaptic plasticity in serotonin receptor-rich brain areas remain entirely unexplored. We applied presynaptic stimuli paired with postsynaptic action potentials (APs) to a subpopulation of CLA neurons projecting to ACC in male rats to find that the psychedelic drug, 2,5-dimethoxy-4-iodoamphetamine (DOI), reverses the polarity of synaptic plasticity from long-term depression (LTD) to long-term potentiation (LTP) in a manner that may reflect contribution of excitatory or inhibitory neurotransmission but is specific to synapses activated by local electrical stimulation. Additionally, we characterize intrinsic electrophysiological properties of CLA–ACC neurons with and without DOI application, noting several changes to AP dynamics induced by DOI. These findings align with the view that psychedelics induce rapid and lasting synaptic plasticity and strengthen the hypothesis that claustrocortical circuits are highly sensitive to psychedelic drug action.

## Significance Statement

Psychedelics are showing promise for treatment of various psychiatric disorders. How do psychedelics promote long-term therapeutic changes in the brain? A leading theory is that lasting neuronal plasticity is induced by psychedelic drug action at 5-HT2Rs. Here, we evaluate neurons in the claustrum, a region with the highest density of 5-HT2Rs in the brain. We report that the psychedelic, 2,5-dimethoxy-4-iodoamphetamine, provokes a net change in synaptic efficacy that manifests as long-term potentiation of excitatory postsynaptic potentials instead of the long-term depression observed under control conditions. These results provide a possible cellular excitability basis of long-term psychedelic drug action.

## Introduction

Synaptic plasticity refers to the brain's ability to adapt and modify the strength of connections between neurons based on experiences. Long-lasting changes in synaptic plasticity underlie the ability for animals to learn and adapt to environmental changes. As changes in synaptic plasticity continue to be a hallmark of diverse psychiatric disease states such as substance use disorder, depression, anxiety, and obsessive–compulsive disorder (Welch et al., 2007; Luscher and Malenka, 2011; Duman and Aghajanian, 2012; Appelbaum et al., 2023), approaches to precisely target plasticity at affected synapses may hold potential as therapeutics. One form of long-term plasticity can be achieved by temporally close pre- and postsynaptic action potentials (APs) as encapsulated by the Hebbian learning theory and observed across species from insects to humans (Caporale and Dan, 2008).

Synaptic release of neuromodulators, such as serotonin (5-HT), has been shown to influence the development of synaptic plasticity within some neurocircuits (Brzosko et al., 2019). Endogenous neuromodulators and clinically used drugs are able to shape the rules that govern the direction and strength of synaptic plasticity. For example, activation of 5-HT4 receptors induces long-term depression (LTD) when postsynaptic stimulation is followed by presynaptic activity in striatal cells (Cavaccini et al., 2018). Similarly, disease states such as depression or substance use disorder that are known to involve serotonergic dysfunction can disrupt synaptic plasticity (Luscher and Malenka, 2011; Duman and Aghajanian, 2012; Appelbaum et al., 2023). Relevant to drug-induced synaptic plasticity, repeated exposure to cocaine has been found to broaden the interval between pre- and postsynaptic stimuli that result in long-term potentiation (LTP) in Layer 5 pyramidal neurons of the prefrontal cortex (Ruan and Yao, 2017). Drugs may also influence metaplasticity or plasticity that depends on prior history of synaptic or cellular changes (Abraham, 2008). Indeed, ketamine, a dissociative *N*-methyl-d-aspartate receptor antagonist clinically used for treatment-resistant depression, has recently been shown to prime neurons in the CA1 region of the hippocampus to the second ketamine exposure that produced significantly greater LTP than the first ketamine administration (Ma et al., 2025). Notably, therapeutic efficacy of ketamine may depend on cellular mechanisms similar to serotonergic psychedelics, with specific roles proposed for rapamycin complex 1 (mTORC1) signaling, γ-aminobutyric acid (GABA_A_) receptor activation, opioid receptor, and inflammatory signals (Johnston et al., 2023). Furthermore, ketamine increases extracellular 5-HT in the prefrontal cortex (Lopez-Gil et al., 2019), and 5-HT depletion blocks the antidepressant effects of (*S*)-ketamine (du Jardin et al., 2017), highlighting a potential role of serotonergic signaling in the effects of rapid-acting antidepressants.

Over the past decade, a resurgence of research on psychedelics has produced promising results for the treatment of psychiatric disease, including depression, anxiety, and substance use disorders, in humans (Yehuda and Lehrner, 2023). Psychedelics act primarily via activation of serotonin receptors, particularly the 5-HT2AR, though other 5-HTRs, such as the 5-HT1A and 5-HT2C, are also activated (Vollenweider and Preller, 2020; Cameron et al., 2023a). A prominent theory is that psychedelics may achieve their lasting therapeutic effects through induction of neuronal plasticity in brain circuits enriched with 5-HT2ARs. Psychedelics induce rapid structural, synaptic, and epigenomic changes in neurons (Jones et al., 2009; de la Fuente Revenga et al., 2021; Shao et al., 2021; Jefferson et al., 2023) and promote lasting changes in functional connectivity across 5-HT2AR-dense brain regions in patients successfully treated for treatment-resistant depression (Daws et al., 2022). Furthermore, several psychedelic, empathogenic, oneirogenic, and dissociative compounds such as psilocybin, lysergic acid diethylamide, 3,4-methylenedioxymethamphetamine, ibogaine, and ketamine have recently been shown to reopen a juvenile-like critical period for social reward learning in adult mice (Nardou et al., 2023). These behavioral effects were accompanied by oxytocin-dependent metaplasticity as excitatory signaling in the nucleus accumbens medium spiny neurons was increased in response to oxytocin 2 weeks after drug administration. These findings give insight into how psychedelics and other psychoactive compounds may harness alterations of synaptic strength to achieve their therapeutic effects, necessitating further investigation into specific neurocircuitry that may be altered by these drugs.

The claustrum (CLA) is a thin nucleus located between the striatum and the insula that has been shown to have among the highest 5-HT2A and 5-HT2CR expression in the brain (McKenna and Saavedra, 1987; McKenna et al., 1990; Anderson et al., 2024). The CLA, the most densely interconnected brain structure by volume (Torgerson et al., 2015), has been proposed as a site for integration of cortical signals due to bidirectional connectivity with multiple regions, particularly the anterior cingulate cortex (ACC; Atlan et al., 2017; Wang et al., 2017; Chia et al., 2020). These observations position the CLA at the center of serotonin receptor-dependent effects on cortical function. However, the effects of psychedelics on long-term synaptic plasticity in the CLA or even the ability of CLA neurons to undergo long-term synaptic changes have never been demonstrated. Since mounting evidence suggests that psychedelics achieve their therapeutic effects by inducing long-term synaptic plasticity (Grieco et al., 2022; Cameron et al., 2023b; Siegel et al., 2024; Shao et al., 2025) and that the CLA is highly responsive to psychedelics (Barrett et al., 2020; Doss et al., 2022; Davoudian et al., 2023; Anderson et al., 2024; Bagdasarian et al., 2024), synaptic plasticity within the CLA circuits may be key to understanding the therapeutic potential of serotonergic psychedelics.

Here, we evaluated long-term plasticity of excitatory postsynaptic potentials (EPSPs) in CLA–ACC neurons with and without the psychedelic drug, 2,5-dimethoxy-4-iodoamphetamine (DOI). We found that the pre–post pairing stimulations associated with synaptic potentiation in other brain areas produced an anti-Hebbian depression of EPSPs in CLA–ACC neurons under control conditions. The expected potentiation, however, was recovered during application of DOI. While DOI effects on interaction between local inhibitory and excitatory synapses remain undetermined, our results highlight the first evidence in support of psychedelic drug ability to control the polarity of long-term plasticity in the CLA–ACC circuit.

## Materials and Methods

### Animals

Male Sprague Dawley rats (*Rattus norvegicus*), weighing 200–250 g, were obtained from Taconic Laboratories. Animals were individually housed, with food and water available *ad libitum* in the home cage. A 12 h light/dark cycle was used with the lights on at 7 A.M. All experimental protocols were approved by the Institutional Animal Care and Use Committee of the University of Kentucky.

### Stereotaxic injections

Rats were anesthetized with isoflurane and CLA–ACC neurons were labeled by the retrograde AAV-hSyn-EGFP (Addgene #50465-AAVrg) injected bilaterally (2 µl/side) into the ACC at the following stereotaxic coordinates (in mm from the bregma), A/P, +0.3; M/L, ±0.9; D/V, −2.2, using a 2 µl Neuros syringe (Hamilton Company) at a rate of 0.2 µl/min.

### Electrophysiology

Brains were rapidly removed, and coronal slices (300 μm thick) containing the CLA were cut using a vibratome (VT1200S; Leica Microsystems) in an ice-cold artificial cerebrospinal fluid (aCSF) cutting solution, containing the following (in mM): 93 NMDG, 2.5 KCl, 1.25 NaH_2_PO_4_, 30 NaHCO_3_, 20 HEPES, 25 glucose, 5 Na-ascorbate, 2 thiourea, 3 Na-pyruvate, 10 MgSO_4_, and 0.5 CaCl_2_, 300–310 mOsm, pH 7.4, when continuously oxygenated with 95% O_2_/5% CO_2_. Slices were allowed to recover in the aCSF cutting solution at 34–36°C for 30 min, during which, increasing volumes of 2 M NaCl (up to a total of 1 ml NaCl/37.5 ml aCSF) were added every 5 min as previously described (Anderson et al., 2024). After recovery, the slices were transferred to a recording aCSF solution maintained at room temperature. Recording aCSF contained the following (in mM): 130 NaCl, 3 KCl, 1.25 NaH_2_PO_4_, 26 NaHCO_3_, 10 glucose, 1 MgCl_2_, and 2 CaCl_2_, pH 7.2–7.4, when saturated with 95% O_2_/5% CO_2_. For electrophysiology recordings, recording pipettes were pulled from borosilicate glass capillaries (World Precision Instruments) to a resistance of 4–7 MΩ when filled with the intracellular solution. The intracellular solution contained the following (in mM): 145 potassium gluconate, 2 MgCl_2_, 2.5 KCl, 2.5 NaCl, 0.1 BAPTA, 10 HEPES, 2 Mg-ATP, and 0.5 GTP-Tris, pH 7.2–7.3 with KOH, osmolarity 280–290 mOsm. CLA–ACC neurons were viewed under an upright microscope (Olympus BX51WI) with infrared differential interference contrast optics and a 40× water-immersion objective. The recording chamber was continuously perfused (1–2 ml/min) with oxygenated recording aCSF warmed to 32 ± 1°C using an automatic temperature controller (Warner Instruments). CLA–ACC neurons were identified by eGFP fluorescence. In DOI experiments, DOI (10 µM) was present in the continuously perfused recording aCSF. All recordings were digitized at 20 kHz and low-pass-filtered at 2 kHz using a Digidata 1550B acquisition board (Molecular Devices) and pClamp11 software (Molecular Devices). Access resistance (10–30 MΩ) was monitored during recordings by injection of 10 mV hyperpolarizing pulses, and data were discarded if access resistance changed >25% over the course of data collection. The stimulation electrode was placed near the external capsule, which is the main white fiber tract for projections to and out of the CLA (Fernandez-Miranda et al., 2008; White et al., 2018) to stimulate presynaptic terminals.

EPSPs were evoked once every 30 s at a stimulation intensity that produced half of the maximal EPSP amplitude (found prior to recording, using a protocol of increasing stimulation intensities to evoke an EPSP every 3 s) using a bipolar tungsten electrode placed between the patched cell and the external capsule of the corpus callosum. The stimulation protocol followed a “pre–post” pairing that is standard for induction of LTP (Ruan and Yao, 2017). A postsynaptic AP was evoked in the patched cell 10 ms after the presynaptic electrode elicited an EPSP. This pairing was repeated at 0.1 Hz for 10 min (60 pairings). Four cells (three in the DOI group and one in the aCSF group) did not survive past 35 min. Intrinsic excitability and AP data from Figure 3 and Tables 1 and 2 were collected by evoking a single AP with a brief 20 ms depolarizing current injection and no prior EPSP. AP threshold was defined as the membrane potential at which the derivative of voltage with respect to time (dV/dt) reaches 10 mV/ms^−1^. AP half-width was calculated as the duration of the spike measured at half of peak amplitude. AP height was defined as the amplitude from threshold to peak, whereas AP amplitude was the amplitude from resting membrane potential (RMP) to the spike peak. The afterhyperpolarization potential (AHP) fast component (within 6 ms of the AP peak) occurred in most recorded CLA–ACC cells, with AHP probability represented as the percentage of cells expressing an AHP. AHP amplitude was measured from the threshold, while AHP latency was measured from the AP peak. The afterdepolarization potential (ADP) was defined as the depolarizing event immediately after the AHP, with ADP probability represented as the percentage of cells expressing an ADP. ADP latency and ADP amplitude were measured from the AHP trough. AP rise time was measured as the time it takes for the membrane potential to go from 10 to 90% of AP height, whereas the AP rise slope was measured as the average rate of voltage change across the same percentile window. The maximum AP rise slope was the largest single instantaneous derivative of the AP trace during the rise to spike peak. AP decay time, decay slope, and maximum decay slope were found using the downward decay of the trace after the spike peak. The RMP was acquired from the average steady-state voltage difference across the neuronal membrane by measuring the average membrane potential across a 500 ms segment of trace free of spontaneous synaptic activity. Input resistance (*R*_in_) and membrane capacitance (*C_m_*) were determined from currents elicited by brief hyperpolarizing voltage pulses (−10 mV). Readers can access all raw data, references to materials used, and associated protocols by request.

**Table 1.** Summary statistics of electrophysiological properties of CLA–ACC neurons in aCSF condition

Condition	AP property	Mean	SEM	Median	SD	*p*
aCSF	AP threshold (mV)	−34.31	1.921	−34.00	6.656	0.1476
aCSF	AP half-width (ms)	1.499	0.0506	1.525	0.1752	0.0150*
aCSF	AP height (mV)	78.18	2.344	78.49	8.121	0.5163
aCSF	AHP fast probability	1.000	0.000	1.000	0.000	0.2301
aCSF	AHP fast latency (ms)	3.755	0.2156	3.485	0.7468	0.0515
aCSF	AHP fast amplitude (mV)	9.383	1.788	7.170	6.193	0.3435
aCSF	ADP probability	0.833	0.1124	1.000	0.3892	0.2457
aCSF	ADP latency (ms)	4.509	1.024	3.695	3.237	0.2257
aCSF	ADP amplitude (mV)	5.229	0.6856	6.225	2.168	0.0415*
aCSF	AP amplitude (mV)	119.6	3.321	120.8	11.50	0.2295
aCSF	AP rise time (ms)	0.4817	0.0176	0.4869	0.0609	0.0132*
aCSF	AP rise slope (mV/ms)	141.6	8.665	131.5	30.02	0.0512
aCSF	Maximum AP rise slope (mV/ms)	168.5	10.30	157.2	35.68	0.0647
aCSF	AP decay time (mS)	1.510	0.0651	1.430	0.2254	0.0416*
aCSF	AP decay slope (mV/ms)	−42.65	1.796	−40.60	6.220	0.0247*
aCSF	Maximum AP decay slope (mV/ms)	−50.46	3.605	−45.47	12.49	0.3832
aCSF	RMP (mV)	−75.02	2.269	−74.31	7.526	0.1186
aCSF	Rin (MΩ)	135.3	31.78	287.4	105.4	0.1346
aCSF	Cm (pF)	133.3	11.22	135.6	37.21	0.5738

*n* = 11 neurons from six rats.

* *p* < 0.05, paired Student's *t* tests of aCSF versus DOI groups.

**Table 2.** Summary statistics of intrinsic electrophysiological properties of CLA–ACC neurons during perfusion of DOI

Condition	AP property	Mean	SEM	Median	SD	*p*
DOI	AP threshold (mV)	−29.29	2.876	−26.66	8.133	0.1476
DOI	AP half-width (ms)	1.866	0.1508	1.825	0.4266	0.0150*
DOI	AP height (mV)	75.15	4.409	74.20	12.47	0.5163
DOI	AHP fast probability	0.8750	0.1250	1.000	0.3536	0.2301
DOI	AHP fast latency (ms)	4.471	0.6655	4.430	0.665	0.0515
DOI	AHP fast amplitude (mV)	12.50	2.875	9.310	7.605	0.3435
DOI	ADP probability	1.000	0.000	1.000	0.000	0.2457
DOI	ADP latency (ms)	6.101	0.5857	5.910	1.657	0.2257
DOI	ADP amplitude (mV)	3.353	0.3931	3.445	1.112	0.0415*
DOI	AP amplitude (mV)	112.7	4.730	114.6	13.38	0.2295
DOI	AP rise time (ms)	0.5959	0.0440	0.5592	0.1244	0.0132*
DOI	AP rise slope (mV/ms)	112.1	11.49	108.7	32.49	0.0512
DOI	Maximum AP rise slope (mV/ms)	134.6	14.38	132.4	40.69	0.0647
DOI	AP decay time (mS)	2.093	0.3142	1.725	0.8887	0.0416*
DOI	AP decay slope (mV/ms)	−33.00	4.040	−35.92	11.43	0.0247*
DOI	Maximum AP decay slope (mV/ms)	−58.52	9.741	−49.44	27.55	0.3832
DOI	RMP (mV)	−70.56	1.475	−69.63	4.171	0.1186
DOI	Rin (MΩ)	215.7	21.75	215.4	61.51	0.1346
DOI	Cm (pF)	123.5	12.81	115.7	36.23	0.5738

*n* = 8 neurons from five rats.

* *p* < 0.05, paired Student's *t* tests of aCSF versus DOI groups are replicated from Table 1 for convenience.

### Data analysis and statistics

**Figure 1.**
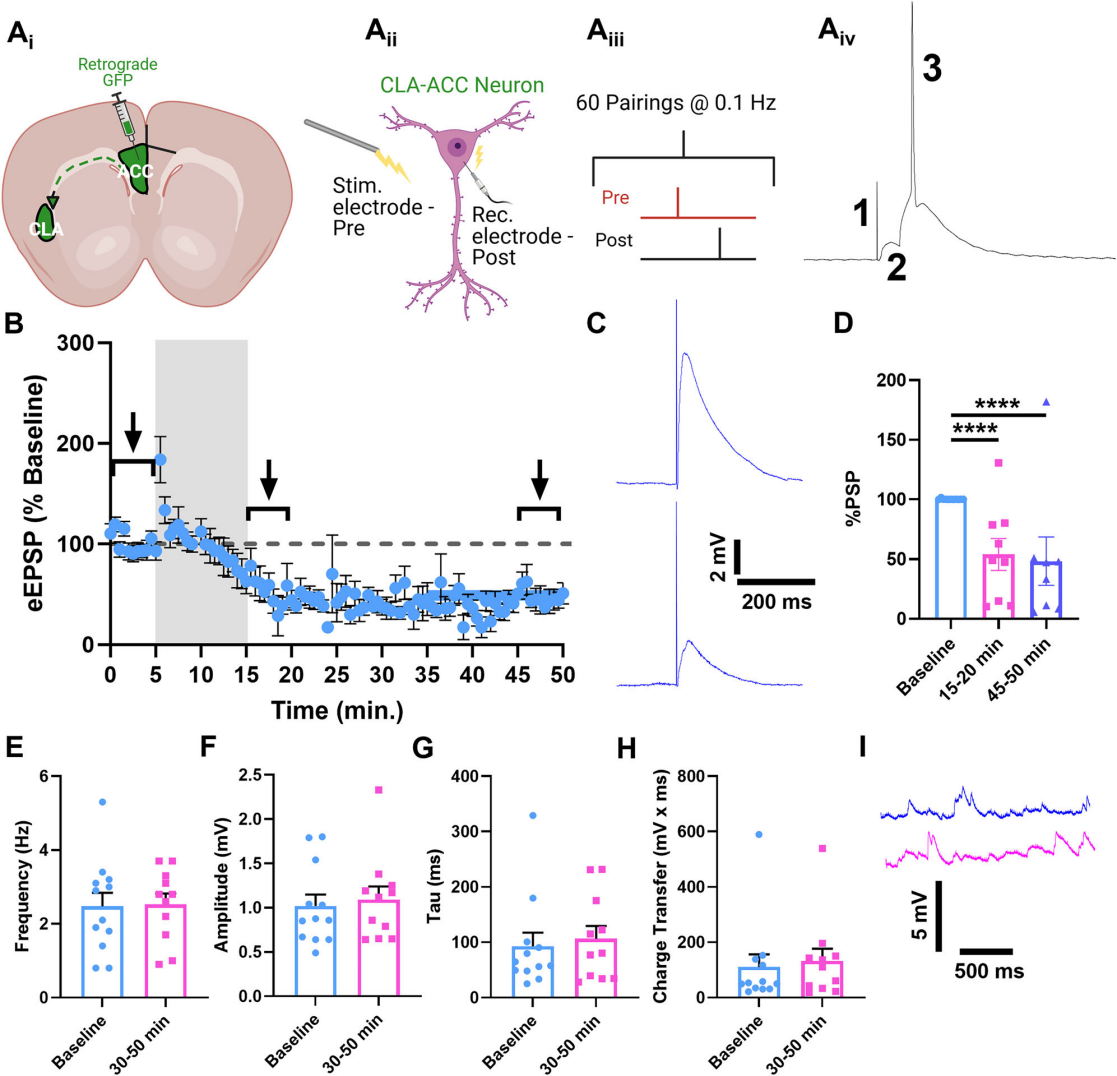
Pre–post stimulation induces LTD in CLA–ACC neurons. ***A***, Graphic of experimental design: ***A*_i_**, CLA–ACC neuron labeling. ***A*_ii_**, Pre–post stimulus pairing. ***A*_iii_**, Stimulation protocol. ***A*_iv_**, A representative CLA neuron response to pre–post stimulation: 1, stimulation artifact; 2, eEPSP; 3, AP. ***B***, Time-course of peak eEPSP amplitudes in all recorded neurons. Gray box indicates the plasticity induction period. Brackets indicate 5 min windows used for preinduction and postinduction analysis in ***D***. ***C***, Representative traces of evoked eEPSPs before (top, 0–5 min) and after (bottom, 45–50 min) plasticity induction. ***D***, Histogram of eEPSP amplitudes as percentage change from the baseline before and after plasticity induction. ***E–H***, Histograms of sEPSP frequency, amplitude, duration (tau), and charge transfer. ***I***, Representative traces of sEPSPs before (blue, 0–5 min) and after (pink, 45–50 min) plasticity induction. ****p < 0.0001, paired Student's *t* tests. *n* = 11 neurons from six rats.

Cells from 5–6 animals were analyzed for electrophysiology experiments in each experimental condition. All analyses were completed using Clampfit 11.1 (Molecular Devices) and Microsoft Excel. Statistical comparisons were performed in Microsoft Excel or GraphPad Prism 10, using two-tailed paired or unpaired Student's *t* tests as indicated. All data were expressed as mean ± SEM. Figure 1*A* graphic was made with Biorender.com.

## Results

### Pre–post pairings induce LTD in CLA–ACC neurons

To evaluate long-term synaptic plasticity in CLA, we identified a subpopulation of CLA neurons projecting to ACC using a retrograde labeling approach (Fig. 1*A*) and targeted these cells for patch-clamp recordings. A stimulating electrode was positioned between the recorded CLA–ACC neuron (50–100 μm) and the external capsule, the main white fiber tract through which the CLA receives predominantly glutamatergic signals from various cortices (Fernandez-Miranda et al., 2008; White et al., 2018). The stimulating electrode delivered a “pre” pulse that evoked a single EPSP (eEPSP) followed 10 ms later by an AP (“post” pulse) induced by depolarization of the patched CLA–ACC cell. Such pre–post pairings are known to produce LTP in many brain regions (Brzosko et al., 2019), in line with the Hebbian postulate that repeated presynaptic stimuli followed by postsynaptic cell activation are necessary for an increase in synaptic strength. However, pre–post pulse pairings in CLA–ACC neurons produced LTD (*t*_(98)_ = 8.627; *p* < 0.0001) that began developing toward the end of the induction period, peaked within 5 min following induction, and was sustained until the end of recording period (*t*_(81)_ = 7.657; *p* < 0.0001; Fig. 1*B*–*D*).

We next evaluated spontaneous EPSPs (sEPSPs) to probe specificity of LTD to electrically stimulated synapses. We found that the sEPSP frequency was not affected by the stimulation (Fig. 1*E*) arguing against a general decrease in synaptic release probability by the pre–post stimulation protocol. Amplitude and duration of sEPSPs were also unchanged, suggesting that the observed LTD was restricted to the population of synaptic events activated by depolarizing “pre” pulses rather than nondiscriminate effects on the entire population of synapses onto the recorded CLA–ACC neurons (Fig. 1*F*–*I*).

### The psychedelic, DOI, reverses the sign of long-term plasticity in CLA–ACC neurons

**Figure 2.**
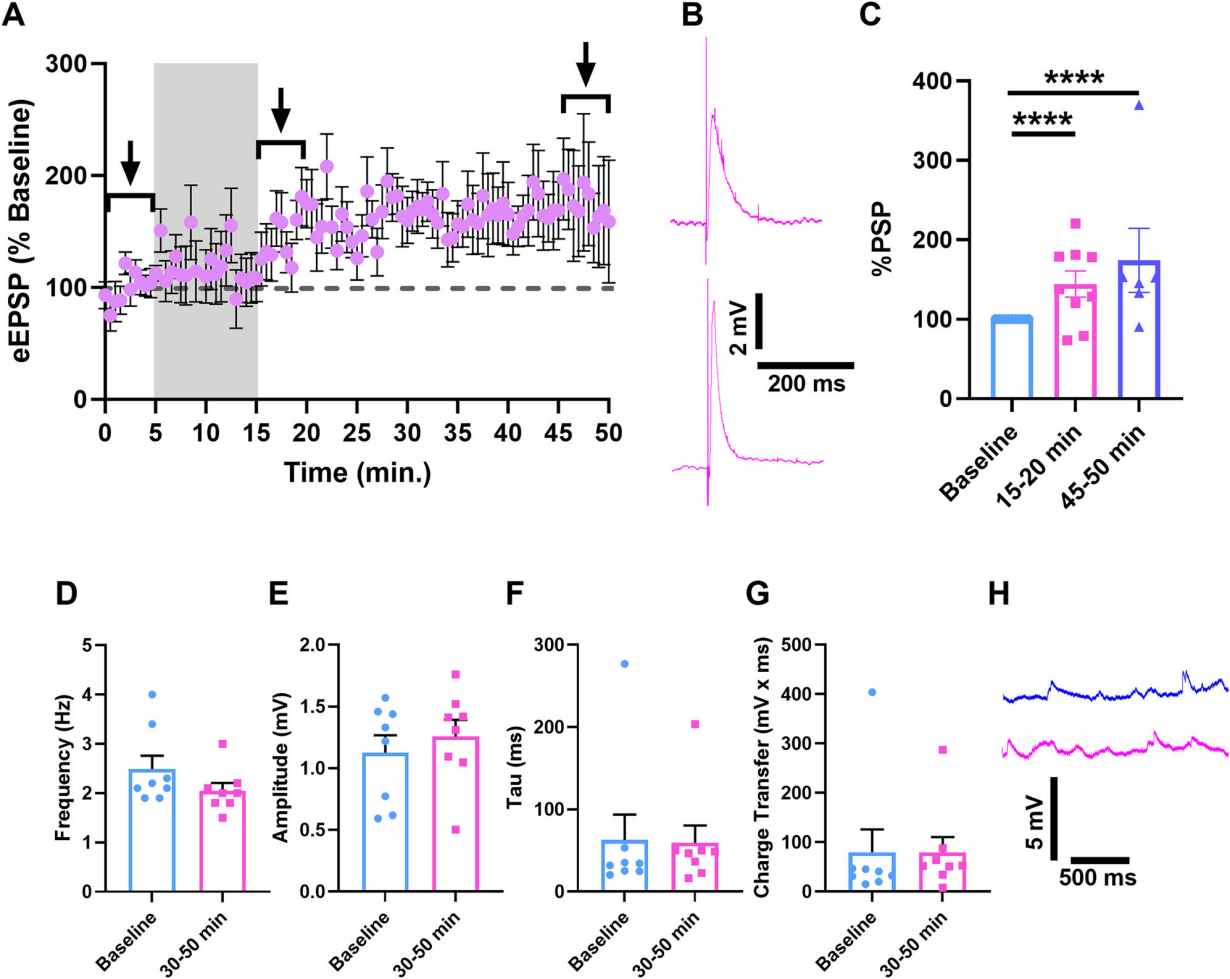
Psychedelic DOI unlocks LTP in CLA–ACC neurons. ***A***, Time-course of peak eEPSP amplitudes in all recorded neurons. Gray box indicates the plasticity induction period. Brackets indicate 5 min windows used for preinduction and postinduction analysis in ***C***. ***B***, Representative traces of eEPSPs before (top, 0–5 min) and after (bottom, 45–50 min) plasticity induction protocol. ***C***, Histogram of eEPSP amplitude as percentage change from the baseline during recording periods indicated by the brackets in ***A***. ***D–G***, Histograms of sEPSP frequency, amplitude, duration (tau), and charge transfer. ***H***, Representative traces of sEPSPs before (blue, 0–5 min) and after (pink, 45–50 min) plasticity induction. ****p < 0.0001; ***p < 0.001; paired Student's *t* tests. *n* = 8 neurons from five rats.

To determine the impact of DOI on long-term synaptic plasticity in the CLA–ACC circuit, we applied identical pre–post stimulation protocols in the presence of bath-applied psychedelic, DOI (10 µM). This manipulation resulted in a robust LTP (*t*_(97)_ = 5.580; *p* < 0.0001) that began to emerge during the 10 min induction period, was markedly increased at cessation of stimulation, and was sustained until the end of the recording (*t*_(65)_ = 5.827; *p* < 0.0001; Fig. 2*A*–*C*). Relative to observations in aCSF, DOI impact on eEPSP amplitude was associated with very large effect sizes: at 15–20 min postinduction Cohen's *d* = 2.01 (power = 0.98) and at 45–50 min postinduction Cohen's *d* = 1.63 (power = 0.79). As in the case of control neurons, sEPSP frequency, duration, and amplitude were all unaffected by the pre–post protocol in the presence of DOI (Fig. 2*D*–*H*), supporting a specific increase of synaptic efficacy at stimulus-activated synapses. Together, these results support an interpretation that CLA–ACC neurons exhibit an anomalous plasticity profile in response to our pre–post plasticity induction protocol and that DOI controls the sign of synaptic efficacy changes during coincident synaptic neurotransmitter release and AP spikes in these cells.

### Effects of DOI on CLA–ACC intrinsic cell properties and AP waveform

**Figure 3.**
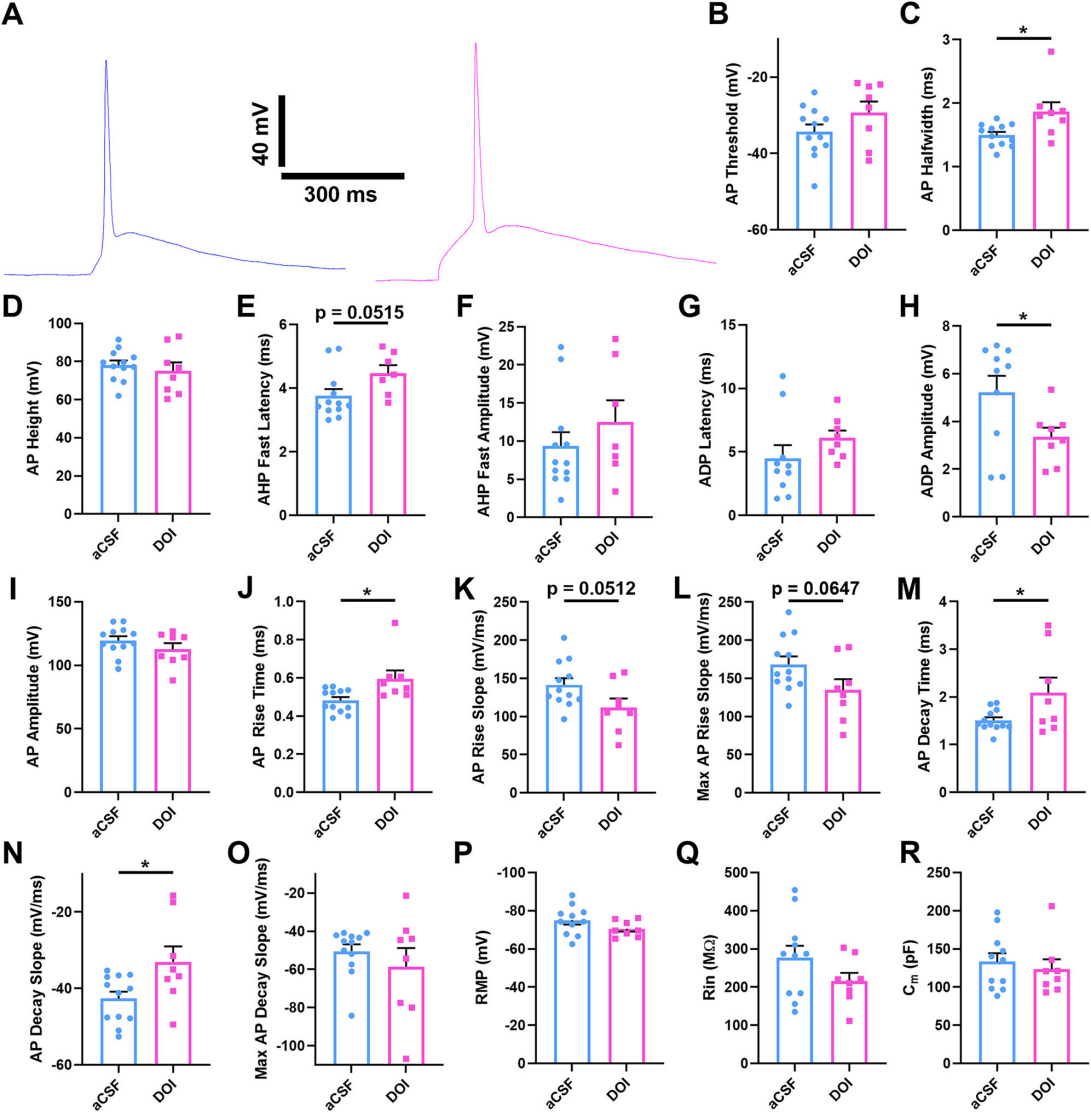
Intrinsic electrophysiological properties of CLA–ACC neurons. ***A***, Representative traces of CLA–ACC APs in regular aCSF (blue) or aCSF with 10 µM DOI (pink). ***B–R***, Histograms comparing AP and intrinsic excitability properties of CLA–ACC neurons in aCSF and DOI conditions. *p < 0.05, paired Student's *t* tests. *n* = 19 neurons from 11 rats.

Evidence suggests that psychedelics may facilitate changes to other electrophysiological measures of excitability aside from synaptic ones, including intrinsic membrane characteristics, likelihood of APs, AP adaptation, and rheobase current (Aghajanian and Lakoski, 1984; Ekins et al., 2023; Anderson et al., 2024; Wang et al., 2025). Some of these effects have been attributed to 5-HT receptor interaction with potassium channels underlying M-currents or G-protein–coupled inward rectifying currents (Ekins et al., 2023; Wong et al., 2024; Wang et al., 2025). We previously found that DOI decreases AP firing in CLA–ACC neurons (Anderson et al., 2024), but did not extensively evaluate changes in AP kinetics. Here, we analyzed data from single APs prior to pre–post pairings with or without DOI to evaluate effects on intrinsic excitability (RMP, *R*_in_, *C_m_*) and the AP waveform. A total of 19 measures were selected for analyses of which five were significantly impacted by DOI (Fig. 3; Tables 1, 2). Specifically, we found that DOI-exposed cells had increased AP half-width (*t*_(17)_ = 2.688; *p* = 0.0150; unpaired Student's *t* test), decreased ADP amplitude (*t*_(15)_ = 2.217; *p* = 0.0415; unpaired Student's *t* test), increased rise time (*t*_(17)_ = 2.749; *p* = 0.0132; unpaired Student's *t* test), increased decay time (*t*_(17)_ = 2.195; *p* = 0.0416; unpaired Student's *t* test), and increased decay slope (*t*_(17)_ = 2.451; *p* = 0.0247; unpaired Student's *t* test). Strong trends that failed to meet the significance threshold in the presence of DOI included elevated latency of fast component of AHP (*t*_(16)_ = 2.095; *p* = 0.0515 unpaired Student's *t* test) as well as decreased AP rise slope (*t*_(17)_ = 2.089; *p* = 0.0512; unpaired Student's *t* test) and maximum rise slope (*t*_(17)_ = 1.968; *p* = 0.0647; unpaired Student's *t* test; Fig. 3; Tables 1, 2). The significant DOI-associated changes followed an overall pattern of an AP that is slower to rise and fall/recover to the baseline, consistent with potassium channel involvement, although the specific identity of the underlying channels remains to be determined.

## Discussion

### Facilitation of LTP in CLA–ACC neurons by psychedelics

Clinical research continues to show promising long-term therapeutic efficacy after a single or a few exposures to psychedelics (Daws et al., 2022; Yehuda and Lehrner, 2023). Such lasting positive changes in behavior suggest psychedelic-induced alterations in neuronal plasticity that may counteract deleterious plasticity frequently associated with psychiatric illness (Appelbaum et al., 2023). Despite increased interest, the neurobiological mechanisms underlying psychedelic-induced long–term synaptic plasticity have been generally understudied. For example, we are aware of only one other investigation of spike-induced plasticity in the presence of psychedelics that reported thalamocortical post–pre LTD was exacerbated by DOI via action at 5-HT2A receptors (Barre et al., 2016). This contrasts with our finding that DOI changes the sign of long-term plasticity in the CLA from depression to potentiation. It is entirely possible that psychedelics promote potentiation at some synapses and depression at others, and, for DOI specifically, we previously observed potentiation of spontaneous excitatory currents in the CLA (Anderson et al., 2024). Related to this, the LTP reported here occurred in continuous presence of DOI both before and after the induction protocol and likely on the background of synaptic potentiation induced by DOI itself (Anderson et al., 2024). Therefore, temporally linked pre- and postsynaptic site stimulation appears to further amplify DOI potentiation of excitatory synaptic strength, providing a potential cellular mechanism for increased salience of interoceptive and exteroceptive events associated with psychedelic experience. Indeed, it has been theorized that some synapses are tagged for plastic changes before a second neuromodulatory step that converts the transient tag into a permanently potentiated or weakened synapse (Gerstner et al., 2018). Such tags have been referred to as “eligibility traces” and evidence of their existence has been found at cortical synapses where conversion of the tag into a measured change in synaptic weight was facilitated by exogenous application or endogenous release of serotonin, dopamine, and norepinephrine (He et al., 2015). We expect that CLA–ACC cells require serotonin receptor activation to generate LTP, though it remains to be seen if endogenous serotonin, other psychedelic drugs, or neuromodulators other than serotonin can also facilitate LTP in this circuit. Eligibility traces may support metaplastic, plasticity of plasticity, events by controlling the window for integration of temporally adjacent stimuli (Abraham and Bear, 1996). An emerging theory surrounding the therapeutic mechanism of psychedelics is that they may facilitate reopening of critical period of plasticity, where established synaptic connectivity is rendered labile, as reported, for example, with social reward learning in mice (Nardou et al., 2023). Our results support the interpretation of psychedelics promoting synaptic changes in CLA–ACC neurons that are distinct from those induced by temporally adjacent pre- and postsynaptic stimulation alone.

### Intrinsic electrical properties and AP kinetics of CLA–ACC neurons

Our analyses of intrinsic cellular properties contribute to the effort of characterizing diversity of CLA neurons based on their electrophysiological signatures. It has previously been reported that CLA neurons that project to cortical regions distribute into subpopulations with distinct intrinsic electrophysiological properties, spiking characteristics, and morphology (White and Mathur, 2018; Graf et al., 2020; Qadir et al., 2022). CLA projection neurons have been classified as “Type 1” neurons that have a lower membrane capacitance, are less likely to burst fire in response to injected depolarizing current steps, have weaker spike adaptation, and have less complex dendritic arbors than “Type 2” neurons (White and Mathur, 2018; Qadir et al., 2022). In other studies, CLA projection neurons have been broken down into five subtypes (PN1–5) based on their intrinsic excitability, AP kinetics, and degree of adaptation during spike trains (Chia et al., 2017; Graf et al., 2020). Our data align most closely with the PN2 and 3 subtypes, based on measures of AP threshold, AP height, AP half-width, maximum AP decay, presence of an AHP and an ADP, as well as AHP amplitude. In the framework of Type 1/2 classification (White and Mathur, 2018; Qadir et al., 2022), our data are consistent with a mixed population of CLA–ACC neurons with six neurons having *C_m_* < 140 pF and five having *C_m_* > 140 pF.

DOI had an overall effect on AP kinetics that was consistent with spikes that were slower to rise and fall, including significant increases in AP half-width and rise time, decreases in AP decay time and decay slope, and a decreased ADP amplitude. As a most parsimonious interpretation, all of these changes could result from DOI interactions with potassium channels. Abundant literature suggests that 5-HTRs interact with potassium currents. Indeed, CLA neuron inhibition by 5-HT has been previously attributed to increased K^+^ ion permeability (Anderson et al., 2024; Wong et al., 2024). In the prefrontal cortex, psychedelics regulate intrinsic excitability of Layer 5 pyramidal neurons via 5-HT2AR activation of M-type potassium currents (Ekins et al., 2023; Wang et al., 2025), often linked to K_v_7.x channel activity. These channels may contribute to the slower AP rise time, as shown for K_v_7.2 (KCNQ2) and K_v_7.3 (KCNQ3) channels (Battefeld et al., 2014). Conversely, blockade of M-currents has been noted to increase burst firing and ADP amplitude in CA1 neurons (Yue and Yaari, 2004). Other types of potassium currents, such as those mediated by K_v_2 (Liu and Bean, 2014), K_v_3 (Rudy and McBain, 2001; Lien and Jonas, 2003), and BK (Sun et al., 2003) channels, also regulate the decay and repolarization of membrane potential following a spike, but their regulation by psychedelic drugs remains to be confirmed. Particularly relevant to Ca^2+^-activated potassium channels, Ca^2+^ ion entry may contribute to some of the effects that we observe. For example, slower repolarization and broader AP have been linked to BK channel activity and presence of extracellular Ca^2+^ in CA1 pyramidal cells of the hippocampus (Shao et al., 1999). Activity of voltage-gated Na^+^ channels may also be involved. For example, altered kinetics of Na_v_ 1.6 or 1.2 has been linked to broader APs and increased AP rise time (Hu et al., 2009). Finally, 5-HT2A and 5-HT2C receptor activation has been shown to increase AP threshold in pyramidal neurons of the prefrontal cortex via protein kinase C (PKC) activation (Carr et al., 2002), and PKC sites are abundant at Na_v_1.2 (Scheuer, 2011) and multiple potassium channels (Cerda and Trimmer, 2010; Gada and Logothetis, 2022).

### Psychedelic action in the CLA

The CLA has been suggested to be central for psychedelic drug action, most prominently in the context of the cortico–claustro–cortical (CCC) model (Doss et al., 2022; Liaw and Augustine, 2023; Bagdasarian et al., 2024). This model builds on anatomical evidence of extensive CCC connectivity to propose that CLA acts as a cortical excitability filter (Mathur, 2014; Jackson et al., 2018; Qadir et al., 2022; Wang et al., 2023). Based on evidence of dense 5-HT2A receptor expression in the CLA and reports of psychedelics markedly impacting CLA activity and functional connectivity with various brain networks, the CCC model further theorizes that disruption of CCC circuits may underlie both the subjective and the long-lasting therapeutic effects of psychedelics (Doss et al., 2022). Our data add mechanistic support for the CCC hypothesis by demonstrating pronounced long-term effects of DOI on synaptic efficacy in CLA–ACC neurons. If psychedelics achieve their therapeutic effects by promoting neuronal plasticity, and if the CCC model of psychedelic action accurately predicts claustrocortical signaling as fundamental to the underlying mechanism of psychedelic long-term and subjective effects, then plasticity induced specifically in CLA–ACC neurons may be key to unifying these theories. Accumulating evidence supports the CLA role in regulation of synchronized brain states and complex behavior (White et al., 2020; Madden et al., 2022; Do et al., 2024) that likely rely on CLA interactions with regions outside the ACC. In that respect, it will be important to investigate whether cellular effects of DOI or other psychedelics vary among CLA neurons in a circuit-specific manner. Of particular interest is the circuit effects and behavioral role of 5-HT2A versus 5-HT2C receptor signaling since both receptor subtypes are activated by psychedelic drugs but with distinct outcomes on intrinsic and synaptic excitability of CLA neurons (Anderson et al., 2024).

### Defining the rules of synaptic plasticity in the CLA

We provide evidence that a 10 ms pre–post pairing protocol activating local synapses onto CLA–ACC neurons results in LTD of EPSPs that is reversed into LTP in the presence of DOI. A number of important questions are raised by this observation. First, what is the role of local inhibition in generating this phenomenon? In this manuscript, we deliberately chose to characterize DOI effects in conditions maximally approaching normal CLA physiology and in the absence of confounding factors from other pharmacological interventions, such as GABA_A_ or potassium channel blockers. We have also previously shown that although ∼20% of neurons within the CLA were GABAergic, local GABA_A_ receptor-mediated tone did not have an effect on serotonin receptor regulation of spontaneous excitatory postsynaptic currents in CLA–ACC neurons (Anderson et al., 2024). Nevertheless, it is possible that extracellular stimulation that we employed here activated both excitatory and inhibitory terminals, despite our efforts to increase contribution of excitatory synapses by placing the stimulating electrode near the external capsule, populated predominantly by excitatory projections to the CLA (Fernandez-Miranda et al., 2008). If our results in regular aCSF were driven by LTP of inhibitory inputs, one could speculate that DOI attenuated inhibition and disinhibited excitatory responses to produce the apparent potentiation that we observed. Such an effect of DOI would be contrary to published data that 5-HT robustly enhances GABA_A_-mediated currents in the rat cortex (Zhou and Hablitz, 1999) and our unpublished observations that exogenous serotonin has a similar potentiating effect on GABAergic transmission in CLA neurons not known to specifically project to ACC. Methodological considerations such as duration of 5-HTR stimulation (Zhou and Hablitz, 1999) may play a role here, as well as interactions between 5-HTRs and potassium channels that may affect GABAergic synapse efficacy (Kasper et al., 2015).

Second, is the anti-Hebbian behavior of CLA–ACC cells maintained across a broader window of pre- and postsynaptic parings outside the 10 ms pre–post interval that we employed? In other words, does CLA–ACC plasticity depend on spike timing interval? A number of studies indicate that serotonin regulates the window for integration of coincident pre- and postsynaptic stimuli. For example, serotonin has been shown to bidirectionally regulate the coincidence window for associative learning in *Drosophila melanogaster* (Zeng et al., 2023), long-term synaptic plasticity in the mollusk *Tritonia diomedea* (Sakurai and Katz, 2003), and gating of LTD induced by post–pre pairings at mouse thalamostriatal synapses (Cavaccini et al., 2018). In our case, the minimal/maximal interval between pre- and poststimuli that maintains LTP in CLA–ACC cells remains to be determined as does the effect of post–pre stimulations. It would further be valuable to investigate whether LTD observed in the absence of DOI is maintained within pairing intervals that are similar to those resulting in LTP when DOI is present. We note that reversal of plasticity rules by neuromodulators is far from unprecedented. Dopamine has been shown to facilitate such reversal in rat corticostriatal synapses (Wickens et al., 1996), mouse hippocampal CA1 neurons (Brzosko et al., 2015), and mouse Layer 5 prefrontal cortex pyramidal neurons (Louth et al., 2021). We have also previously described a mechanism for nicotine receptor-mediated reversal of plasticity rules in the orbitofrontal cortex of the mouse (Zhou et al., 2018). Multiple cellular mechanisms for these effects have been proposed and mechanistic description of psychedelic drug effect on long-term plasticity in CLA–ACC cells should be a goal for future studies. Finally, given mounting evidence describing variability of psychedelic effects across individuals and specific experimental (or environmental) conditions (Roseman et al., 2017; Haijen et al., 2018; Strickland et al., 2021; Woodburn et al., 2024), future studies will need to examine the interaction between behavioral experience and cellular response to psychedelics. In the meantime, our data support the view that psychedelics induce rapid and lasting synaptic plasticity, possibly via metaplastic changes to synaptic strength, and strengthen the hypothesis that claustrocortical circuits are highly sensitive to long-term effect of psychedelic drug administration.

## Synthesis

Reviewing Editor: Mark Sheffield, University of Chicago Division of the Biological Sciences Decisions are customarily a result of the Reviewing Editor and the peer reviewers coming together and discussing their recommendations until a consensus is reached. When revisions are invited, a fact-based synthesis statement explaining their decision and outlining what is needed to prepare a revision will be listed below. The following reviewer(s) agreed to reveal their identity: Alex Kwan, George Augustine.

The manuscript investigates synaptic plasticity in the claustrum and how the psychedelic DOI influences this process. Both reviewers recognize the study's novelty and significance, particularly as it provides the first demonstration of long-term synaptic plasticity in the claustrum and reveals that DOI can reverse the polarity of synaptic plasticity. However, several key concerns must be addressed to ensure the findings are well-supported and methodologically sound.

A primary concern is the lack of clarity regarding the nature of the recorded synaptic inputs. The placement of the stimulating electrode and the potential contribution of inhibitory circuits remain unclear. Without experiments using inhibitory synapse blockers, it is difficult to determine whether the observed LTD reflects suppression of excitatory inputs or enhancement of inhibitory inputs. Additionally, the study does not characterize the intrinsic properties of the recorded neurons, which would be essential for understanding potential cell-type-specific effects.

Another major issue is the limited scope of the study, particularly the single tested time interval for spike-timing-dependent plasticity (STDP). Varying the interstimulus interval would help clarify whether the observed effect represents a shift in plasticity rules or a selective modulation of one specific timing condition. While the results are intriguing, the brevity of the manuscript leaves key methodological and interpretational details underdeveloped.

During the consultation, both reviewers agreed that while the study presents interesting findings, it requires significant revisions and additional discussion to be suitable for publication. The highest priorities should be identifying neuron subtypes, testing STDP across different timing conditions, and examining inhibitory contributions with GABA receptor blockers.

A "Revise and Resubmit" is recommended, as the study has potential merit. However, it is understood that addressing all the comments might take a substantial amount of time. The detailed reviewer comments that follow provide specific guidance for revision.

Reviewer 1:

This study examines the plasticity of ACC-projecting neurons in the claustrum using slice electrophysiology. They show an interesting finding of how the classic psychedelic DOI may change the normally depressing plasticity into a potentiating one under identical protocol. The results though are somewhat preliminary, coming from few cells with various important details missing such as the intrinsic properties and the dependence on pre-post timing.

Comments:

There are related preclinical and clinical studies showing claustrum activity and connectivity being altered by psychedelics (PMID: 36630309; PMID: 32454209; PMID 38916752) that should be cited.

There are also models for how the claustrum may be involved in psychedelic action (e.g., PMID: 34897383).

Is there any rationale for the stimulating electrode placement? One would think the placement is important and can lead to different response and plasticity effects for the recorded neuron.

It is suggested that this is spike timing dependent plasticity but the effect of time is only tested at 10 ms. A mapping of the time range may shed light and show some timing leads to potentiation and other timing leads to depression. It is not clear if the window has shifted or if it is all depressing.

The effect of DOI is interesting, and seems in line with the idea of metaplasticity championed by a few studies (PMID: 37316665).

Was there a characterization of the intrinsic properties of the recorded cell, beyond just the STDP? For instance, membrane potential, threshold to fire, F-I curves, etc. etc.? Those would be useful to know.

Fig. 1D and 2C should show individual data points, so the readers can judge the variability of the results.

Reviewer 2:

This paper shows that the psychedelic compound, DOI, affects long-term synaptic plasticity in the claustrum. The results are notable, both because this is the first demonstration of long-term synaptic plasticity in the claustrum and also because it is the first report (to my knowledge) that a psychedelic can reverse the polarity of long-term synaptic plasticity.

The paper is remarkably short, only 2 figures and combined Introduction/Results/Discussion sections totaling 110 lines of text. Though brevity, in itself, is not a problem, it would have been good for the authors to provide a more critical analysis of their existing data, and perhaps a few more control experiments to make it a more comprehensive study.

My main concern is that the cellular targets of the local stimulation protocol used (Fig. 1Aii) are unclear - are these excitatory inputs from outside the claustrum, activation of local excitatory neurons within the claustrum, or a mixture of excitatory and inhibitory inputs (from local interneurons)? The undershoot on the post-STDP response shown at the bottom of Fig. 1C looks like an IPSP and makes me think that the responses are a poorly-defined mix of multiple types of synaptic input.It is unfortunate that none of the experiments - as far as I could discern from the Methods section - were done in the presence of blockers of inhibitory synapses (e.g. GABA receptor antagonists).

This concern is not an abstract issue, because it is possible that the apparent LTD of excitatory synaptic transmission seen in Fig. 1B instead results from a LTP of inhibitory synaptic transmission. If this is the case, then the actions of DOI shown in Fig. 2 could then result from actions on inhibitory synapses, rather than excitatory synapses.

In summary, the actions of DOI on long-term plasticity in the claustrum are likely to be of interest to many readers of eNeuro. But I encourage the authors to invest more words in discussing what they have measured in their experiments and the potential limitations of their measurements.

## Author Response

Thank you to all reviewers for the enthusiasm about our results and the valuable suggestions. Revisions to address reviewer comments has resulted in a greatly improved manuscript. We have added new analyses, figures, and tables, as well as greatly expanded on the text of the manuscript. We apologize for the delay in producing the revised version, the first author graduated, started his postdoc, and had a child in the months since the original submission. Our point-by-point replies to each reviewer are italicized below:

Reviewer 1:

1- This study examines the plasticity of ACC-projecting neurons in the claustrum using slice electrophysiology. They show an interesting finding of how the classic psychedelic DOI may change the normally depressing plasticity into a potentiating one under identical protocol. The results though are somewhat preliminary, coming from few cells with various important details missing such as the intrinsic properties and the dependence on pre-post timing.

We have analyzed intrinsic properties and actional potential kinetics data from all recorded neurons and formed Figure 3 and two additional Tables to include 19 membrane excitability measures. The number of cells in the aCSF and DOI groups that we were able to collect reflects the challenge of maintaining stable whole-cell recordings during the long duration of spike-timing experiments and is similar or exceeds cell numbers in other published work examining this form of long-term plasticity with patch-clamp electrophysiology. With the existing cell numbers and the large effect size of DOI relative to aCSF condition, the achieved statistical power is 0.98 (Cohen's d=2.01) during 15-20 min post-induction interval and 0.79 (Cohen's d=1.63) during the 45-50 min interval. These calculations boost confidence in our conclusion that DOI reverses the direction of spike timing plasticity in the CLA and are included in the revised manuscript. The pre-post timing comment is discussed in reply to point 4 below.

2- There are related preclinical and clinical studies showing claustrum activity and connectivity being altered by psychedelics (PMID: 36630309; PMID: 32454209; PMID 38916752) that should be cited. There are also models for how the claustrum may be involved in psychedelic action (e.g., PMID: 34897383).

These references have been added to the text, and we have expanded the introduction and discussion sections to address the topics of claustrum activity, connectivity, and relationship to psychedelics.

3- Is there any rationale for the stimulating electrode placement? One would think the placement is important and can lead to different response and plasticity effects for the recorded neuron.

The stimulation electrode was always placed between the patched CLA-ACC neuron and the white matter tract of the external capsule. We found this placement ideal for consistent generation of evoked EPSPs in coronal slices. This makes sense given evidence that the dorsal external capsule consists predominantly of claustrocortical fibers and the bi-directional projections between claustrum and cortex travel through the corpus callosum (PMID: 18377257, 29298436). Unlike cortex, hippocampus, and some other brain areas, the claustrum lacks clear cell layer boundaries, but it is possible that CLA-ACC neurons in different CLA sub-regions or CLA neurons that do not project to the cortex would respond differently to stimuli from different loci of origin within the CLA. Stimulating near the claustrocortical fibers seemed logical for the first evaluation of long-term synaptic plasticity in CLA-ACC cells that we describe in this study. We have added lines to the methods and main text to clarify the stimulation electrode placement and the reasoning behind it.

4- It is suggested that this is spike timing dependent plasticity but the effect of time is only tested at 10 ms. A mapping of the time range may shed light and show some timing leads to potentiation and other timing leads to depression. It is not clear if the window has shifted or if it is all depressing.

An important point. We remain deeply interested in the effects of DOI and psychedelic drugs on duration of 'coincidence/integration windows' defined by the interval between pre- and post-synaptic signals, especially in light of literature showing effects of serotonin and other neuromodulators on such integration windows (PMID: 14645466, 22007168). We agree that our results do not address dependence of plasticity on spike timing and in revised manuscript (including in manuscript title), we use 'spike-timing plasticity' when referring to our experimental outcomes. Additionally, we address the testing of only one pre-post timing interval as a limitation in the revised Discussion.

5- The effect of DOI is interesting, and seems in line with the idea of metaplasticity championed by a few studies (PMID: 37316665).

We agree that the ability of DOI to reverse spike-timing plasticity could be an example of metaplasticity. We have significantly expanded the introduction and discussion to acknowledge this interpretation along with the possibility that DOI may facilitate creation of eligibility traces at CLA-ACC neuron synapses. PMID: 37316665 is now cited among other work on metaplasticity in the revised text.

6- Was there a characterization of the intrinsic properties of the recorded cell, beyond just the STDP? For instance, membrane potential, threshold to fire, F-I curves, etc. etc.? Those would be useful to know.

We analyzed the intrinsic properties of all recorded cells as well as their action potential dynamics and added another figure, two tables, and text to the manuscript to describe these results and highlight the changes in these measures induced by DOI. We would like to thank the reviewer for suggesting these edits that added substantial depth to the manuscript.

7- Fig. 1D and 2C should show individual data points, so the readers can judge the variability of the results.

All figures in the manuscript now show individual data points within the graphs.

Reviewer 2:

1- This paper shows that the psychedelic compound, DOI, affects long-term synaptic plasticity in the claustrum. The results are notable, both because this is the first demonstration of long-term synaptic plasticity in the claustrum and also because it is the first report (to my knowledge) that a psychedelic can reverse the polarity of long-term synaptic plasticity. The paper is remarkably short, only 2 figures and combined Introduction/Results/Discussion sections totaling 110 lines of text. Though brevity, in itself, is not a problem, it would have been good for the authors to provide a more critical analysis of their existing data, and perhaps a few more control experiments to make it a more comprehensive study.

We agree that the original submission was very short. We have added new analysis and greatly expanded introduction, results, and discussion sections which we feel has resulted in a greatly improved, more comprehensive, manuscript.

2- My main concern is that the cellular targets of the local stimulation protocol used (Fig. 1Aii) are unclear - are these excitatory inputs from outside the claustrum, activation of local excitatory neurons within the claustrum, or a mixture of excitatory and inhibitory inputs (from local interneurons)? The undershoot on the post-STDP response shown at the bottom of Fig. 1C looks like an IPSP and makes me think that the responses are a poorly-defined mix of multiple types of synaptic input. It is unfortunate that none of the experiments - as far as I could discern from the Methods section - were done in the presence of blockers of inhibitory synapses (e.g. GABA receptor antagonists).

As the present manuscript was our first attempt to characterize DOI effects on long-term plasticity, we deliberately opted to preserve whatever inhibitory tone may be present at synapses onto CLA-ACC neurons, aiming for a slice preparation that was maximally relevant to normal CLA physiology and reasoning that one of our principal goals was to evaluate DOI effects in the absence of confounding factors from other pharmacological interventions. We acknowledge that the original trace illustrating post-STDP response in Figure 1C contains a clear hyperpolarizing component that could represent a GABAergic IPSP or, perhaps, activation of a voltage-gated potassium conductance. As it happens, this was the only record that showed such a component and we replaced the trace with a more representative example. We added a mention of our published observation that local inhibitory (GABAA) tone has no effect on serotonin receptor regulation of spontaneous excitatory post-synaptic currents in CLA-ACC neurons (PMID: 39218140). Local electrical stimulation, as in the present manuscript, is clearly more likely to activate inhibitory and excitatory terminals simultaneously than spontaneous neurotransmitter release. If our results in regular aCSF were driven by LTP of inhibitory inputs, one could surmise that DOI attenuates inhibition and disinhibits excitatory responses to produce the apparent potentiation that we report. This effect of DOI, however, would be contrary to published data that 5-HT robustly enhances GABAA-mediated currents in the rat cortex (10601434) and our unpublished data of similar 5-HT effect in CLA neurons that were not specifically known to project to ACC. Methodological issues such as duration of 5-HT receptor stimulation may be at play (10601434) along with likely interaction between 5-HT receptor subtypes and potassium ion channels previously raised by us (39218140) and others (8389838). These considerations are included in the revised discussion.

3- This concern is not an abstract issue, because it is possible that the apparent LTD of excitatory synaptic transmission seen in Fig. 1B instead results from a LTP of inhibitory synaptic transmission. If this is the case, then the actions of DOI shown in Fig. 2 could then result from actions on inhibitory synapses, rather than excitatory synapses? Please see our reply to point 2 above. We whole-heartedly agree that this is an important issue.

4- In summary, the actions of DOI on long-term plasticity in the claustrum are likely to be of interest to many readers of eNeuro. But I encourage the authors to invest more words in discussing what they have measured in their experiments and the potential limitations of their measurements.

We appreciate the thoughtful review. We have made significant edits to the manuscript, expanding the introduction, results, methods, and discussion section to better highlight relevant background information, data regarding the intrinsic/action potential properties of the patched CLA-ACC neurons, and interpretation of our findings along with inevitable limitations and need for further experimentation.
